# Supplementary material for: Dynamic Transcriptome Analysis Reveals Transcription Factors Involved in the Synthesis of Ethyl Acetate in Aroma-Producing Yeast
Source: Genes (Basel). 2022 Dec 11;13(12):2341. doi: 10.3390/genes13122341 (PMC9777979; doi:10.3390/genes13122341)
Supplement: Supplementary file 1 [file genes-13-02341-s001.zip › genes-2037973-supplementary.pdf]

Supplementary Information

Dynamic transcriptome analysis reveals transcription factors involved in the synthesis of ethyl acetate in aroma-producing yeast

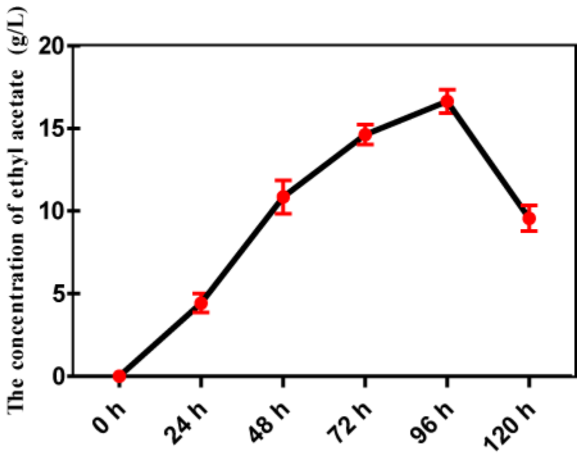

Figure S1 The changes of ethyl acetate content during different periods.

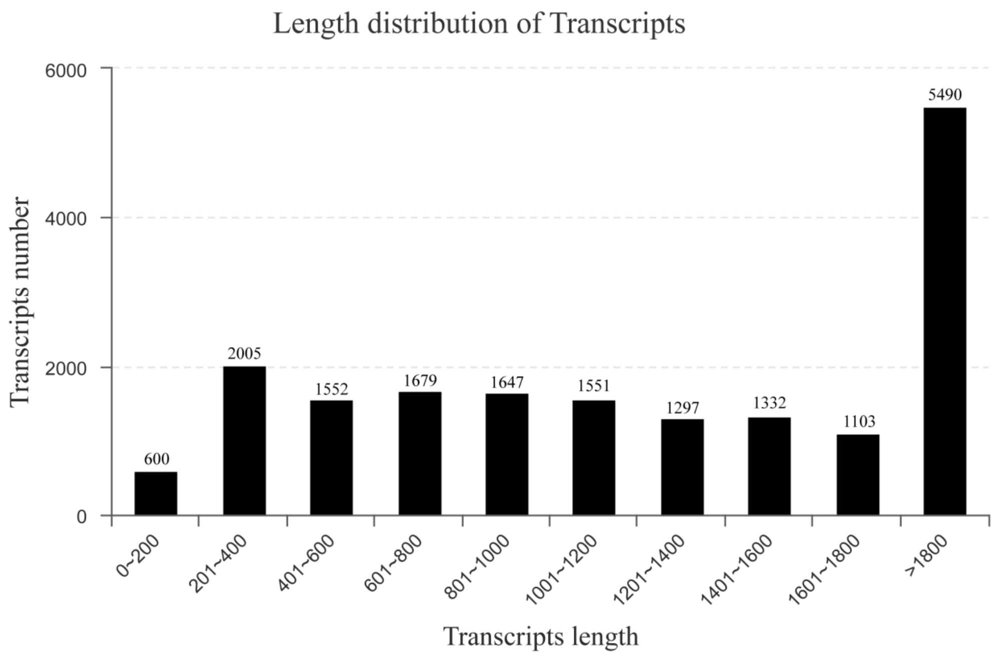

Figure S2 Length distribution of the transcripts length.

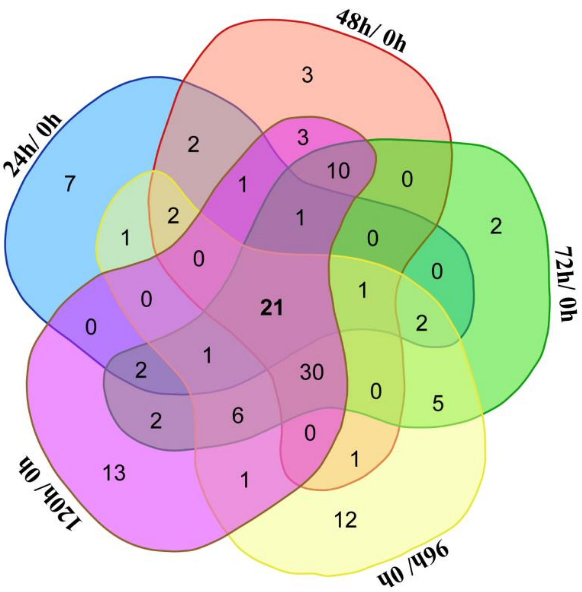

Figure S3 Venn diagram of TF genes under different fermentation time. Venn diagrams showing the total number of significantly TFs (p-value ≥0.05) at 24 h, 48 h, 72 h, 96 h and 120 h fermentation time compared to 0 h.

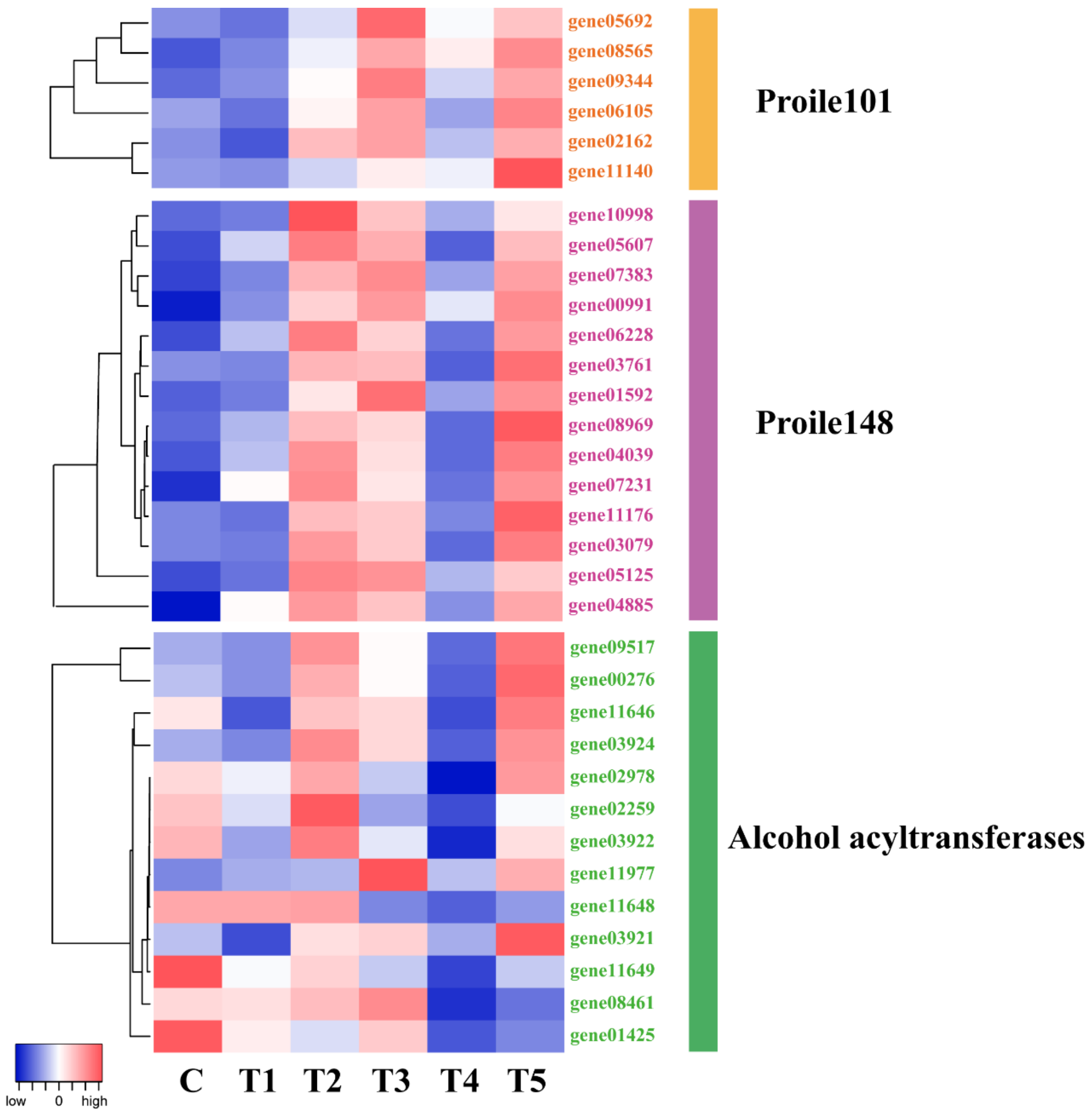

Figure S4 Heat map of expression profiles of TF genes in Proile148, Proile101 and alcohol acyltransferases in *W. anomalus* strain YF1503. The color of the bar represents the level of each transcript's expression abundance, with blue being a low level and red indicating a high one.

Table S1 Primers of 12 TF genes used for real-time quantitative PCR.

**For gene**	**Primer F (5'-3')**	**Primer R (5'-3')**
gene00732	TCAAAATTGGCCAAGAGCTGG	AACATCTTCTTCAACGCTCCAAT
gene09989	TGATGCGATTGATGCTAGGTT	TATTGCTGCTGCTGACCTGA
gene01129	CATCACTCAAAGTGCGCCAT	CACCAAAGACTGGAAAAGCAGG
gene01175	AGACAAGGGCAATAACTTGACT	ACGTCTGAAACTGCAACGGA
gene08213	TTGCCGTTTCAGGCGTTTCTAT	CAGAACTCGAAGGACTACCGA
gene01273	GCAAGCTGATGGTGTAGGTG	ACAGCATACAGGTACCTCCAAA
gene08309	AAATACGCTGCTGCTAAGGC	AACACCAACACCATCAGCTTG
gene07385	CACCAATTTCGGCCAAACAC	AATTTCTACACCTGAAGCTTGTTTA
gene08565	GAATTGTCTGTTGCCGGTGG	CACCATTCGCGACCAGAAAG
gene02199	TGGCTTTAGCTTTGGGTGTTTC	GAAGAAACACAAAGAGGGTCG
gene04039	AGAAGGCTTTAGAGTTCCAAGT	GGAAAGAAAGTGTCAACGGCA
gene04885	TACAGGATTGAGCGGCGAAG	GGCCAAAACAACTACACCACA
ACT1	ATGCTGGTTTCGCTTTAC	AGCAGTAGTGGTGAATGAA

Table S2 Summary statistics of functional annotation for YF1503 unigenes in public databases

Public protein database	Gene number	Percent (%)
GO	10751	80.89
KEGG	7211	54.25
COG	8261	62.15
NR	12571	94.58
Swiss-Prot	10495	78.96
Pfam	10928	82.22
Total_anno	12599	94.79
Total	13291	100

Table S3 Summary statistics of seven main possible model profiles recognized by STEM.

Proile	Gene id	description	C	T1	T2	T3	T4	T5	Proile	Gene id	description	C	T1	T2	T3	T4	T5
	gene00740	zf-GATA	0	-1.75	-1.12	-1.46	-2.27	-1.81		gene00822	MYB	0	-0.41	-0.72	-0.31	-2.15	-1.17
	gene01139	zf-GATA	0	-0.75	-1.08	-1.48	-2.51	-1.4		gene01028	MYB	0	-1.02	-0.9	-1.46	-2.57	-1.08
	gene04349	zf-GATA	0	-1.97	-1.25	-2.39	-3.19	-1.89		gene04262	MYB	0	-2.3	-1.55	-1.68	-2.73	-2.08
	gene12283	zf-GATA	0	-1.67	-0.74	-1.04	-2.35	-1.02		gene09037	MYB	0	-2.59	-1.49	-1.78	-2.78	-1.81
	gene01093	zf-C2H2	0	-1.34	-1.27	-1.69	-2.59	-1.4		gene10077	MYB	0	-1.21	-1.23	-0.97	-2.42	-1.32
Proile16	gene01248	zf-C2H2	0	-1.3	-0.78	-1.23	-2.48	-1.24	Proile16	gene11744	MYB	0	-0.72	-1.1	-1	-3.42	-1.32
	gene02371	zf-C2H2	0	-1.78	-0.99	-0.96	-2.12	-0.82		gene00662	Histone-like TF	0	-1.36	-0.76	-0.83	-2.6	-0.53
	gene04286	zf-C2H2	0	-1.23	-0.75	-0.66	-1.34	-0.64		gene02277	Histone-like TF	0	-1.99	-2.77	-2.89	-3.76	-2.83
	gene07032	zf-C2H2	0	-1.41	-0.7	-1.08	-2.84	-0.65		gene08200	Histone-like TF	0	-2.42	-1.48	-1.56	-3.82	-0.24
	gene07975	zf-C2H2	0	-2.83	-3.52	-4.17	-6.45	-3.97		gene05943	GATA	0	-1.77	-0.95	-1.3	-2.65	-1.29
	gene08106	zf-C2H2	0	-2.54	-1.36	-2.72	-5.29	-1.21		gene09123	GATA	0	-1.62	-1.89	-2.37	-3.98	-2.14

	gene08284	zf-C2H2	0	-1.51	-0.75	-1.23	-2.61	-1		gene01245	Fork head	0	-0.62	-0.83	-0.9	-1.72	-0.74
	gene09061	zf-C2H2	0	-1.1	-0.78	-0.97	-2.41	-1.1		gene05339	CRF1	0	-1.12	-1.36	-1.78	-4.75	-1.27
	gene10443	zf-C2H2	0	-1.39	-1.38	-2.21	-3.49	-1.68		gene06938	CRF1	0	-1.34	-0.85	-1.61	-3.62	-1.07
	gene10843	zf-C2H2	0	-3.47	-4.51	-3.94	-6.66	-4.75		gene02441	bZIP	0	-2.18	-1.83	-2.04	-3.46	-0.58
	gene11431	zf-C2H2	0	-1.14	-0.78	-1.32	-1.77	-1.08		gene04986	bHLH	0	-2.17	-1.34	-2.3	-4.3	-2.07
	gene00142	VHR1	0	-2.78	-1.9	-2.89	-4.39	-2.84		gene07287	bHLH	0	-2.25	-1.16	-1.96	-3.08	-1.47
	gene09645	VHR1	0	-2.53	-1.84	-3.27	-5.32	-3.07		gene04772	ARA1	0	-1.52	-1.9	-0.98	-2.81	-1.2
Proile32	gene03239	zf-GATA	0	-1.2	-0.04	-0.78	-2.17	-0.93	Proile32	gene01007	MYB	0	-0.35	0.22	-0.26	-0.83	-0.4
	gene01065	zf-C2H2	0	-2.29	-1	-2.55	-4.66	-1.4		gene01755	Homeobox	0	-1.23	0.22	-0.68	-3.04	-0.71
	gene02919	zf-C2H2	0	-1.98	-0.82	-1.94	-3.65	-1.18		gene03480	Homeobox	0	-0.97	0.07	-1.28	-3.1	-0.49
	gene05246	zf-C2H2	0	-1.74	-0.59	-1.91	-3.98	-0.88		gene01997	GATA	0	-1.3	-0.35	-0.83	-2.38	-0.82
	gene10791	zf-C2H2	0	-1.07	-0.63	-1.49	-2.73	-1.22		gene01271	C3H	0	-1.69	-0.36	-3.27	-6.1	-1.58
	gene01330	TFIIS	0	-0.7	0.4	-0.89	-2.2	-0.14		gene00583	bZIP	0	-2.07	-0.45	-1.37	-3.09	-0.94
	gene09844	TFIIB	0	-1.58	-0.51	-0.74	-2.33	-0.84		gene05621	bZIP	0	-0.61	0.4	-1.32	-3.33	-1.1
	gene11476	TFG	0	-2.24	0.23	-1.55	-2.68	-1.29		gene06242	bZIP	0	-1.52	0.27	-1.4	-4.01	-1.15
	gene06402	PC4	0	-0.79	-0.11	-0.59	-2.3	-0.54		gene03957	BFR2	0	-2.53	0.23	-3.52	-4.5	-2.13
	gene00784	MYB	0	-1.26	-0.21	-0.92	-2.39	-0.64		gene12011	BFR2	0	-2.72	0.16	-2.83	-4.74	-1.25
Proile148	gene05607	ZBTB	0	1.03	1.87	1.67	0.27	1.61	Proile148	gene10998	Homeobox	0	-0.07	1.57	1.31	-0.4	1.73
	gene06228	ZBTB	0	1.11	2.2	1.77	0.48	2.09		gene03761	GATA	0	1.53	5.85	5.11	3.47	4.75
	gene07231	TFIIIC	0	2.81	3.52	2.95	1.44	3.49		gene08969	GATA	0	-0.09	1.19	1.14	-0.38	1.49
	gene04885	TFIIH	0	2.39	2.94	2.75	1.73	2.89		gene11176	GATA	0	0.33	0.93	0.8	-0.01	1.25
	gene05125	TFIID	0	0.27	1.46	1.4	0.6	1.16		gene00991	bHLH	0	-0.07	0.82	0.75	0.02	1.13
	gene04039	TFC	0	2.13	3.56	3.04	0.63	3.67		gene01592	bHLH	0	0.65	1.25	1.47	0.97	1.52
	gene07383	STE	0	0.44	1.37	1.54	0.58	1.45		Gene03079	RFX	0	0.53	2.02	2.7	1.09	2.52
Proile0	gene11678	zf-MIZ	0	-0.5	-0.64	-1.09	-1.72	-2.17	Proile0	gene03018	HSF	0	-0.15	-0.78	-0.88	-1.11	-1.71
	gene08133	zf-C2H2	0	-1.07	-1.48	-1.82	-2.76	-2.19		gene05272	HMG	0	-0.37	-1.05	-1.73	-2.75	-3.34
	gene12788	zf-C2H2	0	1.83	-2.37	-3.1	-4.61	-6.05		gene07007	HMG	0	-0.06	-0.95	-1.66	-2.44	-3.24
	gene07404	ZBTB	0	-0.06	-0.18	-0.58	-1.13	-1.41		gene10314	GATA	0	0.26	-0.57	-1.03	-1.69	-2.5
Proile101	gene06105	TFIIB	0	-0.57	0.9	1.46	0.07	1.6	Proile101	gene02162	RFX	0	-0.83	1.32	1.46	0.46	1.39
	gene08565	SRF	0	0.98	2.18	2.94	2.47	3.11		gene05692	MTEF1	0	-0.23	0.65	1.73	0.86	1.25
	gene09344	SRF	0	0.45	1.41	2.13	1.1	1.93		gene11140	C3H	0	-0.91	1.3	2.21	1.81	3.43
Proile41	gene04253	zf-MIZ	0	-0.9	0.08	-0.22	-1.52	-0.22	Proile41	gene06713	HMG	0	-1.52	1.21	-0.15	-2.7	0.98
	gene01480	zf-C2H2	0	-1.08	-0.34	-0.35	-0.9	-0.59		gene03147	Histone-like TF	0	-3.62	0.59	-0.64	-3.44	0.45
	gene06703	zf-C2H2	0	-2.04	-1.04	-0.37	-1.58	-0.64		gene01296	GATA	0	-2.77	-1	-1.11	-3.34	-0.3
	gene08535	zf-C2H2	0	-2.36	0.03	-0.34	-1.94	0.39		gene08330	GATA	0	-2.97	-0.75	-1.15	-3.22	-0.42
	gene09377	zf-C2H2	0	-1.94	-0.21	-0.78	-3.14	-0.05		gene02831	C3H	0	-2	-0.18	-0.91	-1.86	-0.58
	gene10596	zf-C2H2	0	-2.06	-0.26	-0.9	-2.2	-0.3		gene04418	C3H	0	-1.77	-0.14	0.06	-1.49	-0.64
	gene12192	zf-C2H2	0	-1.96	-0.26	-0.22	-2.33	-0.51		gene04698	C3H	0	-0.32	0.88	0.57	-0.79	1.06
	gene02047	ZBTB	0	-0.48	0.61	0.64	-1.25	0.66		gene05702	C3H	0	-1.55	-0.26	-0.5	-1.78	-0.62
	gene04867	ZBTB	0	-1.29	-0.03	-0.52	-1.6	0.58		gene05846	C3H	0	-2.44	-0.6	-0.41	-2.89	0.22
	gene09904	TFIIS	0	-2.71	0.34	-1.35	-3.83	0.08		gene06810	C3H	0	-2.17	-1.01	-0.51	-1.99	-0.02
	gene05584	TFIID	0	-1.52	-0.53	-0.08	-1.76	0.51		gene09187	C3H	0	-2.08	-0.68	-0.55	-1.65	-1.02
	gene00591	TFIIB	0	-1.44	-0.57	-0.45	-1.8	-0.71		gene11141	C3H	0	-0.47	0.86	0.56	-0.75	0.95

	gene12611	SUS1	0	-3.15	-0.13	-1.15	-2.63	0.13		gene00874	bZIP	0	-3.63	-0.48	-0.95	-2.01	0.45
	gene02671	PC4	0	-1.24	-0.32	-0.68	-2.24	-0.14		gene09836	bZIP	0	-1.36	0.05	-0.71	-2.19	-0.1
	gene10040	MYB	0	-1.37	-0.52	-0.68	-2.17	-0.29		gene03518	bHLH	0	-2.03	0.73	0.32	-1.62	-0.21
	gene10997	Homeobox	0	-1.49	-0.71	-0.49	-1.12	-0.53		gene06736	bHLH	0	-1.27	0.6	0.32	-2.7	0.59
	gene05747	HMG	0	-1.77	1.05	-0.04	-2.36	0.65	Proile70	gene02924	ZBTB	0	-0.83	-0.92	-1.95	-2.85	-1.89
Proile70	gene04606	HMG	0	-0.91	-0.89	-1.87	-2.76	-1.79		gene08368	TFIIS	0	-0.23	0.33	-0.97	-2.3	-0.43
	gene07006	HMG	0	-0.05	-0.53	-0.65	-2.28	-1.23		gene01257	TFIIIC	0	-1.18	-1.11	-2.46	-4.23	-2.92
	gene11837	FAR1	0	-0.08	-0.32	-0.47	-1.73	-0.85		gene08293	TFIIIC	0	-1.37	-1.34	-2.68	-4.33	-3.11
	gene08307	C3H	0	-1.71	-0.76	-3.89	-8.17	-2.6		gene03830	MYB	0	-0.84	-0.9	-1.41	-4.07	-1.59
	gene09306	bHLH	0	-0.41	-1.19	-2.57	-3.73	-1.96		gene04094	Homeobox	0	0.13	0.14	-0.56	-0.99	-0.29

Table S4 Summary statistics of information on the alcohol acyltransferase member of *W. anomalus strain* YF1503

**Gene ID**	**Swiss-Prot_description**	**FPKM values of gene in samples**																	
		**C_1**	**C_2**	**C_3**	**T24_1**	**T24_2**	**T24_3**	**T48_1**	**T48_2**	**T48_3**	**T72_1**	**T72_2**	**T72_3**	**T96_1**	**T96_2**	**T96_3**	**T120_1**	**T120_2**	**T120_3**
gene00276	Ethanol acetyltransferase	101.02	60.42	172.97	94.9	128.71	1.31	448.54	67.69	230.08	130.48	194.17	157.01	44.15	41.33	18.52	407.45	329.88	236.34
gene09517	Ethanol acetyltransferase	78.9	46.55	161.55	98.31	107.99	0.9	556.9	61.03	300.1	149.95	189.98	193.6	47.26	45.4	19.65	450.42	313.4	276.3
gene03924	Alcohol O-acetyltransferase	30.46	38.92	31.56	39.33	34.04	7.86	108.42	27.99	67.39	44.96	60.45	52.3	23.26	26.89	14.89	92.74	45.09	64.6
gene11977	Alcohol O-acetyltransferase	0.07	0.76	1.02	0.39	1.23	0.44	0.72	0.92	0.52	0.82	0.75	2.28	0.79	1.11	0.29	1.01	0.76	1.38
gene11646	Alcohol O-acetyltransferase	52	65.97	76.91	47.51	37.17	11.35	111.43	35.31	70.7	58.88	74.69	68.67	36.3	38.01	16.06	116.93	64.7	82.96
gene03922	Alcohol O-acetyltransferase	1.39	1.14	0.91	0.58	0.91	0.17	2.37	0.67	1.13	0.61	0.78	0.89	0.35	0.18	0	1.22	1.21	0.44
gene11648	Alcohol O-acetyltransferase	1.48	2.15	1.98	2.27	2.37	1.03	3.27	1.15	1.44	0.42	0.32	0.3	0.31	0.14	0	0.82	0.29	0.31
gene03921	Alcohol O-acetyltransferase	1.92	6.98	3.11	2.26	2.81	1.05	7.03	7.1	3.69	5.51	8.09	5.57	3.68	4.12	3.12	8.39	10.09	9.33
gene11649	Alcohol O-acetyltransferase	1.87	8.06	12.66	6.93	5.72	1.33	6.99	5.07	4.39	4.56	5.6	2.2	1.96	3.84	1.56	4.06	4.01	4.15
gene02259	Alcohol O-acetyltransferase	1.29	0.76	0.14	0.59	0.96	0	1.49	0.48	0.97	1	0.05	0.2	0	0.27	0.5	1.34	0.05	0.33
gene02978	Alcohol O-acetyltransferase	0.81	1.47	0.49	1.07	1.3	0	1.78	0.34	1.01	0.48	0.56	1.1	0.4	0.55	0.14	1.24	0.98	1.05
gene08461	Abhydrolase	37.32	61.28	73.41	71.56	65.66	31.58	62.24	65.57	52.27	83.09	70.32	41.44	48.6	54.27	13.05	37.33	60.61	33.03
gene01425	Abhydrolas	45.01	92.78	98.12	68.05	69.15	43.11	60.43	62.88	42.68	74.51	80.71	37.47	50.79	62.25	15.64	38.23	68.41	33.59
